# Childhood Lead Exposure from Battery Recycling in Vietnam

**DOI:** 10.1155/2015/193715

**Published:** 2015-10-26

**Authors:** William E. Daniell, Lo Van Tung, Ryan M. Wallace, Deborah J. Havens, Catherine J. Karr, Nguyen Bich Diep, Gerry A. Croteau, Nancy J. Beaudet, Nguyen Duy Bao

**Affiliations:** ^1^Department of Environmental & Occupational Health Sciences, University of Washington, Box 357234, Seattle, WA 98195, USA; ^2^National Institute of Occupational and Environmental Health, 57 Le Quy Don, Hai Ba Trung, Hanoi, Vietnam; ^3^School of Medicine, University of Washington, Box 356340, Seattle, WA 98195, USA; ^4^Department of Psychiatry, Yale School of Medicine, 300 George Street, Suite 901, New Haven, CT 06511, USA; ^5^Liverpool School of Tropical Medicine, Karonga Prevention Study, P.O. Box 46, Chilumba, Karonga District, Malawi; ^6^Department of Environmental & Occupational Health Sciences and Department of Pediatrics, University of Washington, Box 354695, Seattle, WA 98195, USA; ^7^Department of Environmental & Occupational Health Sciences, University of Washington, Box 354695, Seattle, WA 98195, USA; ^8^Department of Medicine, University of Washington, Box 359739, Seattle, WA 98195, USA

## Abstract

*Background*. Battery recycling facilities in developing countries can cause community lead exposure. *Objective*. To evaluate child lead exposure in a Vietnam battery recycling craft village after efforts to shift home-based recycling outside the village. *Methods*. This cross-sectional study evaluated 109 children in Dong Mai village, using blood lead level (BLL) measurement, parent interview, and household observation. Blood samples were analyzed with a LeadCare II field instrument; highest BLLs (≥45 *μ*g/dL) were retested by laboratory analysis. Surface and soil lead were measured at 11 households and a school with X-ray fluorescence analyzer. *Results*. All children had high BLLs; 28% had BLL ≥45 *μ*g/dL. Younger age, family recycling, and outside brick surfaces were associated with higher BLL. Surface and soil lead levels were high at all tested homes, even with no recycling history. Laboratory BLLs were lower than LeadCare BLLs, in 24 retested children. *Discussion*. In spite of improvements, lead exposure was still substantial and probably associated with continued home-based recycling, legacy contamination, and workplace take-home exposure pathways. There is a need for effective strategies to manage lead exposure from battery recycling in craft villages. These reported BLL values should be interpreted cautiously, although the observed field-laboratory discordance may reflect bias in laboratory results.

## 1. Introduction

The global demand for lead has risen as much as tenfold in the past decade, mostly linked to the battery industry [1–5]. The most rapid growth has occurred in developing countries, which commonly rely on recycling used lead-acid batteries (ULABs) to meet their demand for lead [6]. In Vietnam, it is projected that over 70,000 tons of ULABs will be reprocessed in 2015, nearly twice the 40,000 tons reprocessed in 2010 [5, 7].

While lead recycling has flourished, controlling the associated occupational and environmental health hazards is problematic [8]. Workplace and environmental regulations are often nonexistent, weak, or poorly enforced in developing countries, particularly outside of formal work settings. Lead recycling operations often consist of informal secondary smelters run by low-income individuals or households, with few or no safety precautions or environmental controls [5, 6, 9]. Typical operations involve manually breaking whole batteries, separating metal and plastic components, melting lead in open vats, casting into ingots, and selling to brokers or battery manufacturers. One report in 2012 estimated the global burden of disease from industrial pollutants to be 17.1 million disability-adjusted life years (DALYs) lost [10]. ULAB recycling accounted for 28% of that disease burden.

Children may face substantial health risk in such situations. A 2011 literature review found that children living near battery manufacturing and recycling facilities had a mean blood lead level (BLL) of 29 *μ*g/dL, in ten studies from seven developing countries [11]. In Senegal, at least 18 children died from rapidly progressive neurologic disease associated with neighborhood ULAB recycling operations; fifty surviving children had BLLs ranging from 39.8 to 613.9 *μ*g/dL [12]. For comparison, the geometric mean BLL for US children is 1.5 *μ*g/dL [13], and the reference level for public health intervention in the USA is 5 *μ*g/dL [14, 15]. The US Centers for Disease Control and Prevention (CDC) recommends chelation treatment if a child's BLL is ≥45 *μ*g/dL.

In 1986 Vietnam launched the “Doi Moi” economic renovations to facilitate transition from a planned economy to a socialist-oriented market economy [16]. Many “craft” villages generated employment and income in rural areas by focusing village activity on a traditional handicraft or activities like metalworking, leather tanning, or metal or plastic recycling. Recent studies in two Vietnamese craft villages specializing in battery recycling demonstrate the potential for lead contamination and human intoxication in these settings [17, 18].

Dong Mai village, in Hung Yen province in northern Vietnam (Van Lam district and Chi Dao commune; population reported by commune officials as 2600 people in 637 households), has been a ULAB recycling center since the 1980s. A study in Dong Mai in 2006-07 by the Vietnam National Institute of Occupational and Environmental Health (NIOEH) found elevated environmental and child urine lead levels [19]. Subsequently, Dong Mai attempted to shift home-based recycling to an industrial zone one kilometer outside the village, with cooperative and private operations. The zone was formalized in 2010, and a large number but not all household ULAB operations had moved by the time of the present study.

The objective of the present study, begun in 2011, was to determine if child lead exposure was still a problem in Dong Mai and—after the study detected disturbing levels of child lead intoxication—to identify likely exposure sources and management options. Note that the findings of a separate study conducted in Dong Mai during 2007–11 were not known to Vietnam NIOEH or University of Washington (UW) researchers, nor Dong Mai village or commune officials, until a 2014 journal publication [17]. That study reported a median BLL of 29 *μ*g/dL (range 17–48) in 16 children and higher BLLs in concurrently tested adult men (median 43, range 23–122, *n* = 30) and adult women (median 36, range 14–87, *n* = 40).

## 2. Materials and Methods

### 2.1. Overview

This cross-sectional study evaluated children in Dong Mai village, aged ten years or less, for possible lead exposure and intoxication using child BLL measurements, parent interview, and household observations. This was a collaboration by Vietnam NIOEH and UW, as part of a Children's Environmental Health Research Training Initiative with paired bilateral trainees (Tung and Wallace) and mentors (Diep, Karr, and Daniell) [20]. Initial field work was conducted in December 2011, including staff from Hung Yen Provincial Preventative Medicine Center and the Commune Health Center for Dong Mai village. The study was extended in December, 2012, to convene a stakeholder workshop and conduct surface and soil lead measurements. All study procedures were approved by institutional review boards at NIOEH and UW.

### 2.2. Training

NIOEH field researchers were trained on proper aseptic blood collection technique and use of the LeadCare II Blood Lead Test System (Magellan Diagnostics). An educational presentation and discussion highlighted the significance of lead poisoning, current US CDC recommendations for diagnosis and management of lead poisoning (note that there are no Vietnamese or international guidelines, although World Health Organization-invited experts' reports are consistent with CDC recommendations) [14, 15, 21–23], and case studies from other countries that have had childhood lead exposure problems. An educational session for commune health workers highlighted the significance of potential elevated BLLs and what the community could do to reduce child lead exposure.

### 2.3. Study Sample

All children, 10 years of age or younger, in Dong Mai village were eligible (no exclusions). Commune health leaders provided a list of approximately 300 eligible children, and 120 were selected randomly for invitation. A recruitment team consisting of NIOEH researchers and provincial and commune health staff visited family homes to invite participation. The researchers described the study, its voluntary nature, and a monetary incentive ($10 USD for each child, at time of blood sampling). Interested families completed an adult consent and child assent process. The resultant study sample included 109 children (91% participation) in 82 households: 56 households with 1 participating child; 25 with 2 children; and 1 with 3 children.

### 2.4. Questionnaire and Observations

At each participating household, a parent or guardian completed an interviewer-administered questionnaire with questions about the child, current or past home-based or other lead recycling by family members, proximity to current or past recycling activity, and household features. A team member completed an observation form about floor and yard surfaces, presence of a garden, and use of battery casings. The family was given an appointment during the following weekend for blood collection.

### 2.5. Blood Collection

Families arrived at the Chi Dao Commune Health Center during one-hour long appointment periods, each accommodating about 20 children. Commune health workers gave instructions on handwashing. Each child used a wash station with three serial wash basins: scrubbing, soap application and removal, and rinsing. Many children also rinsed their hands with tap water before using the wash station. Each child's age, sex, height (or length), and weight were recorded. Blood was collected by fingerstick: the finger was cleaned with an alcohol swab; a new sterile lancet was used to penetrate a fingertip; the first drop of blood was wiped off with a clean and dry swab; a 50 *μ*L blood sample was collected with a capillary tube; and the sample was placed in a LeadCare reagent tube.

### 2.6. Blood Analysis

Blood samples were analyzed with the LeadCare instrument that same day, during or after the blood collection session, at the Chi Dao Commune Health Center (Dong Mai Clinic), in ambient conditions within the LeadCare recommended range (54–97°F and 12–80% relative humidity) [24, 25]. This instrument uses an electrochemical technique (anodic stripping voltammetry) to measure blood concentration after blood cell lysis with a dilute hydrochloric acid reagent. The instrument was calibrated before each new lot of test supplies (every 48 tests), and standard controls were run to assess accuracy. Limits of quantitation are 3.5 to 65 *μ*g/dL. Lower or higher values yield “low” or “high” readings. The manufacturer reported that precision (coefficient of variation) is 12.1% for BLLs in the lower quantifiable range (average BLL 5.3 *μ*g/dL), 7.6% and 5.5% for BLLs in the intermediate quantifiable range (averages 11.0 and 22.9 *μ*g/dL, resp.), and 3.5% for higher quantifiable BLLs (average 51.7 *μ*g/dL) [24]. Manufacturer reported that accuracy relative to analysis by Graphite Furnace Atomic Absorption Spectrometry (GFAAS) is +0.07 *μ*g/dL bias for BLLs 0–10 *μ*g/dL, +4.7% for BLLs 10.1–25.0 *μ*g/dL, and +5.0% for BLLs 25.1–65 *μ*g/dL [24]. A clinical study with untrained operators found that >95% of samples were within the allowable tolerance of error, relative to GFAAS analysis, and based on US Occupational Safety and Health Administration (OSHA) test proficiency recommendations (GFAAS ±6 *μ*g/dL for BLLs ≤40 *μ*g/dL; GFAAS ±15% for BLLs >40 *μ*g/dL) [24]. The average bias was only +1.9% for 64 samples with BLLs 40.1–65.0 *μ*g/dL.

Confirmatory retesting was recommended for (and was conducted for 24 of 31) children with BLL ≥45 *μ*g/dL, with a venipuncture blood sample and BLL analysis at the NIOEH laboratory using mixed nitric/perchloric acid digestion (modified NIOSH method 7300) followed by analysis with GFAAS (NIOSH method 7082) [26, 27]. NIOEH laboratory protocol includes daily calibration with four standards (1–40 *μ*g/dL; agreement <5%) and intermittent duplicate-sample analysis (2-3 per 20 samples; agreement <5%). The protocol does not include certified reference materials, but analyses of spiked acidified aqueous samples (2 and 100 *μ*g/dL) must yield >80% recovery; usual recovery is about 90%.

### 2.7. Reporting

Test results were reported to each child's parent by commune health workers on the day of sample collection, with oral and written interpretation of results, recommendations for treatment (if indicated, per CDC guidelines), and information about lead exposure reduction. All families received a picture-based educational pamphlet describing simple ways to reduce child lead exposures (wet mopping and dusting, mat at door entry, removing shoes and work clothes before entering home, hand washing, discouraging child play in soil, and healthy diet ensuring iron, vitamin C, and calcium). All but seven children had BLL ≥20 *μ*g/dL (minimum 12 *μ*g/dL); therefore, the team advised all families to remove all possible sources of lead exposure, emphasizing personal and household hygiene.

For 31 children with BLL ≥45 *μ*g/dL, the head of the Commune Health Center and a village health worker immediately notified families, emphasized the need to eliminate lead exposures or ensure that children are kept away from current or past ULAB recycling locations or work garments, and recommended confirmatory retesting (24 children participated). Clinical evaluation was recommended to identify symptoms or signs of lead toxicity that might warrant urgent treatment or hospitalization. Retesting cost was covered by the study. Evaluation or treatment was covered by insurance (universal, under age 6; otherwise, family insurance).

The research team had no access to confidential medical information. By anecdotal report from commune representatives, a few children received chelation treatment, while most did not because of limited parent awareness and the distance between the village and treatment facility (Bach Mai hospital in Hanoi, a 1-2-hour drive each way by personal vehicle). Chelation treatment would have had limited and possible worsening value in the face of continuing, prevalent lead exposure [21]. Recommendations for exposure control were prioritized. However, implementation of those recommendations was constrained by limited exposure data, economic pressures against changing or relocating recycling activities, political challenges, and resource constraints.

Therefore, to help residents and authorities answer exposure questions and address health concerns prompted by the study findings, the study was extended to conduct a preliminary assessment of lead surface and soil contamination and to convene a stakeholder workshop.

A half-day stakeholder workshop was held in December, 2012, hosted by Vietnam NIOEH and UW, with invitations to local, provincial, and national government representatives; regional healthcare and academic institutions; nongovernment organizations (NGOs) interested in environmental health; and international organizations. Presentations described study findings, global ULAB experience, and remediation efforts in other Vietnam metalworking craft villages. Small and large group discussions considered barriers, opportunities, and resources to reduce lead exposure in Dong Mai.

### 2.8. Surface and Soil Sampling

Using a Bruker (S1 Turbo) hand-held energy dispersive X-ray fluorescence (XRF) analyzer, a total of 193 surface and 27 soil measurements were conducted over two days in December, 2012, at an elementary school and a convenience sample of three types of homes: active recycling (*n* = 3), past recycling (*n* = 4), and no history of recycling (*n* = 4). In general, at each home three measurements were taken on the ground or floor in each room or major space (middle of space, 1 measurement; sides of space, 2). Some additional measurements were made on above ground, horizontal-surface household items. The XRF device was operated in “paint” mode for surface samples (lower detection limit, 0.1 *μ*g/cm^2^) and “soil” mode for soil samples (lower detection limit, 1 mg/kg), with measurements over a 30-second period. Prior to field use, the device was tested with five NIST surface standards (US National Institute of Standards and Technology; 0, 0.24, 0.88, 1.4, and 3.4 mg/cm^2^).

### 2.9. Data Analysis

All analyses used Stata (version 12.1), SPSS (version 19), or Microsoft Excel. Children were treated as independent units of analysis, other than analyses of possible within-household influences.

BLL was treated as a categorical variable in most analyses, because measurements beyond the LeadCare instrument's limit of quantitation (>65 *μ*g/dL) were reported only as “high.” BLL was categorized based on CDC recommendations for treatment (≥45 *μ*g/dL) and two categories of similar sample size for lower values (10–29.9 and 30–44.9 *μ*g/dL).

Age was categorized relative to school-enrollment age (6–10 years) and two groups of similar sample size for children under six (0–2 and 3–5 years). Height or length was transformed to age- and sex-adjusted, percent predicted values and compared to the 5th lower percentile, using US CDC charts [28]. Surface and soil lead concentrations were only analyzed descriptively because of the limited number of tested households.

Bivariate analyses between categorized BLL and independent variables were conducted first for all children (without considering within-household influences) and then separately for younger (0–5 years) and older (6–10) children, after initial analysis revealed a strong association between age and BLL. Analyses used chi-square or Fisher exact tests (categorical variables) or one-way ANOVA (numeric variables).

Multivariate analyses of BLL used multinomial logistic regression. The multinomial approach allowed us to analyze BLL as a three-category dependent (outcome) variable and identify whether independent variables might have different degrees of association with either or both of the two higher BLL categories (30–44.9 and ≥45 *μ*g/dL). Independent variables that showed statistically significant associations with BLL in the bivariate analyses were examined one at a time for statistical significance in the regression model. Variables were retained in the model based on strongest additional contribution to the model (significance, change in pseudo *R* ^2^) and goodness of fit. Two variables, home recycling and proximity to recycling facility, could not be considered for the model because of the small number of affected households. Possible between-covariate interactions were considered. There were no substantial interactions between variables included in the final model. Then other independent variables that did not show significance in bivariate analyses were added individually to the finalized model to test for associations that might not have appeared in bivariate analysis.

Children from the same household might have similar conditions that influence their BLL. If so, they would not be truly independent subjects, and the household rather than the child should be the unit of statistical analysis. Possible within-household influences were assessed several ways. First, bivariate analysis was repeated using only the youngest and then only the oldest child from each multiple-child household, and results were examined for substantial departures from the analysis using all children. Second, the multinomial logistic regression controlled for within-household influences. Third, within-household concordance between the BLLs of the youngest and oldest child was examined for all households containing more than 1 child.

Relationships between the LeadCare measurements and the GFAAS laboratory analysis—for the 24 subjects with confirmatory retesting—were characterized by graphic visualization and the distribution of differences between paired values.

## 3. Results

### 3.1. Subjects

About half of the 109 participating children were male (47%). The mean age was 4.5 years (standard deviation [SD] 2.6; range 11 months to 10 years). The mean body mass index (BMI) was 15 kg/m^2^ (SD 1.8; range 9.8–20.7). The mean height or length was 96% of (US) age- and sex-predicted values (range 84–110%).

### 3.2. Households

Four children lived in three (out of 82) households where recycling activities took place at the home, in spite of village efforts to restrict this practice. In addition, 60 children (55%) had family members involved in recycling at a centralized facility. More than half of the subjects (62%) lived within 100 m from a current or past recycling site, and one-quarter (25%) lived within 100 m from where recycling waste was or had been burned. Most households had finished yard surfaces (e.g., brick or cement; 87%), but about half had gardens (46%). Half of households had soil floors (50%), usually covered at least partly with mats (46%). Old battery casings were used as containers or stacked as barriers or supports inside and/or outside the home at about half of households. Reported and observed battery casing use were closely similar. There was no substantial difference between age groups in any of the recycling proximity or household variables, except for living within 100 m from waste burning, which occurred most often in the oldest age group.

### 3.3. Blood Lead Levels

**Figure 1:**
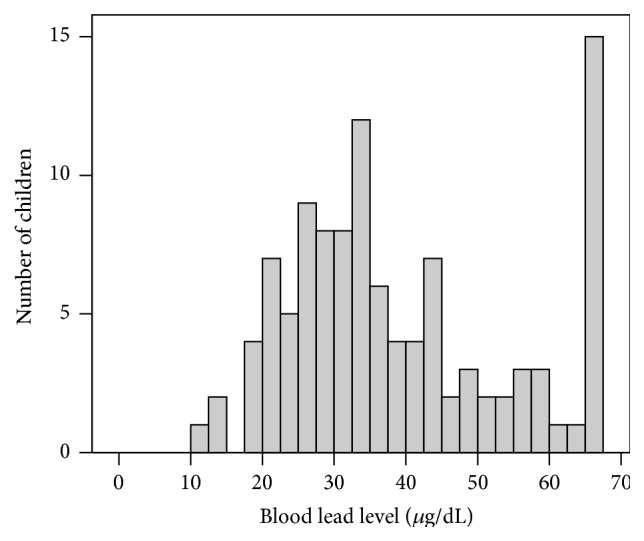
Distribution of child blood lead levels (*n* = 109). Blood was collected by fingerstick and analyzed with LeadCare II instrument. Upper limit of detection, 65 *μ*g/dL.

All children in the study had high BLLs, ranging from 12 to >65 *μ*g/dL (upper limit of LeadCare quantitation). The BLLs showed a bimodal distribution (Figure 1), with one mode at about 25–35 *μ*g/dL, and 15 children having BLL >65 *μ*g/dL.

Most children (24 of 31) whose initial BLL was ≥45 *μ*g/dL had a venipuncture sample collection and laboratory (GFAAS) analysis. In general, the retest BLLs were lower than on initial LeadCare tests. Of 15 initial BLL values that exceeded the 65 *μ*g/dL upper limit of measurement on initial testing, only two were that high on laboratory retesting (65 and 74 *μ*g/dL); the 13 other above-limit initial BLLs were lower on laboratory retesting (mean 52.3, SD 7.1) although still clinically excessive. Among nine other children with quantifiable BLL values on initial testing, the retest BLLs were generally lower (mean difference −11.2, SD 18) with a wide range of differences versus initial values (−41 to +22). Only five retested children (20%) had a BLL <45 *μ*g/dL on retesting (minimum, 26.3 *μ*g/d).

### 3.4. Bivariate Analysis

We examined variables possibly associated with relatively higher BLL, while remaining cognizant that* all* BLLs in this study sample were high. BLL showed no association with symptoms, physical growth, or sex (Table 1). Age was inversely associated with higher BLL (chi-square for trend, *p* = 0.01). BLLs were higher with current recycling activities at the home, which affected four children in three homes. In contrast, child BLLs were not significantly higher at households where home-based recycling occurred in the past, regardless of type of activity. Current (but not past) family involvement in recycling was significantly associated with very high BLL (chi-square for trend, *p* = 0.002). Use of brick in the yard showed a suggestive association with relatively higher BLL.

### 3.5. Multivariate Analysis

Three variables were associated with higher BLL in the multinomial logistic regression model (Table 2): younger age, current family involvement in centralized or home-based recycling, and brick yard surface. Home-based recycling was associated with BLL in bivariate analyses but could not be included in the model because of the small numbers of children affected (i.e., zero values). The multinomial model controlled for possible within-household influences.

### 3.6. Within-Household Influence

Within-household influences appeared minimal, justifying use of each child rather than households as the unit of analysis. There were 56 households with 1 participating child; 25, with 2 children; and 1, with 3 children. When bivariate analyses were repeated using only the oldest child and then only the youngest child in each household, the results did not appreciably differ. At the 26 households with two or more children, the youngest child generally had a BLL in the same category (65%) or one to two BLL categories higher (23% and 4%, resp.) than the oldest tested child. The youngest child had a lower BLL at only 2 households (8%).

### 3.7. Surface Lead

Surface lead concentrations were very high in all 11 sampled homes, regardless of recycling history. The overall mean surface lead concentration of 95 *μ*g/cm^2^ was 2,375 times the US EPA standard for dust on household floors (40 *μ*g/foot^2^ or 0.043 *μ*g/cm^2^) [29]. Surface lead was below XRF detection in 12% of samples (0.1 *μ*g/cm^2^; treated as 0.05 in mean calculations). The detection limit is 2.5 times greater than the US regulatory level; therefore, some nondetected samples might exceed this level. The mean surface lead level at three homes with active recycling (250 *μ*g/cm^2^) was 3.3 times higher than the four homes with past recycling (86 *μ*g/cm^2^); and the mean level at the four past recycling homes was 1.4 times higher than the four homes with no recycling history (60 *μ*g/cm^2^). At homes with no active recycling, the highest surface levels were in washing areas (mean 156 *μ*g/cm^2^) and some kitchen and living areas (mean 50 *μ*g/cm^2^). The mean lead concentration for brick surfaces (141 *μ*g/cm^2^) was about twice that for concrete and tile surfaces (70 and 78 *μ*g/cm^2^, resp.), possibly reflecting bricks' porosity; this difference was less evident at homes with no recycling history. Work clothes tested at one past recycling home were markedly contaminated (6 samples; mean 250 *μ*g/cm^2^).

The mean surface lead level at the school, 41 *μ*g/cm^2^, was less than half that in the tested homes. However, this was still 1,000 times greater than the US EPA limit for lead on household floors. Notably, the highest levels at the school were on four sleeping mats (mean 221 *μ*g/cm^2^).

### 3.8. Soil Lead

None of the 27 soil samples could be collected from active recycling homes. Nearly two-thirds (64%) of samples exceeded the US EPA standard for soil in a children's play area (400 mg/kg) [29]. The highest levels were in four areas where battery recycling was previously conducted, mean about 2,500 mg/kg, about twice the US EPA limit for* non*play areas (1,200 mg/kg). Soil lead levels in yard areas where battery recycling was not performed were lower but still high, mean about 1,000 mg/kg. Nine samples at the school were very low, mean 34 mg/kg, with the highest value being <20% of the US EPA standard for a children's play area.

## 4. Discussion

The blood lead levels of all children screened in this battery recycling village were high, many of them substantial. This occurred in spite of exposure control efforts that began in 2006 to centralize recycling activity outside the village and restrict home-based recycling. Although recycling within the village declined over time, it did persist.

The specific values of BLLs reported here should be interpreted cautiously, because of the discrepancy observed between field instrument and GFAAS BLL measurements, in the subgroup of children with highest field measurements (BLL ≥45 *μ*g/dL) and confirmatory retesting. In this study, the BLL values obtained by fingerstick samples and LeadCare field instrument analysis were generally higher than values obtained soon thereafter by venipuncture and laboratory GFAAS analysis (mean difference 11.2 *μ*g/dL; excluding unquantifiable “high” LeadCare measurements, >65 *μ*g/dL). However, there is as at least as much reason to be concerned about the study GFAAS measurements, as the LeadCare measurements.

The LeadCare instrument is well established as a reliable instrument for nontraditional laboratory settings. It was accurate compared to GFAAS in a manufacturer controlled study and in a clinical study involving independent untrained operators, producing minimal upward bias (2–5%) for higher BLLs, and meeting US OSHA recommendations for tolerance of error [24]. The instrument is recognized by the US Food and Drug Administration as acceptable (“CLIA-waived”) for nontraditional laboratory settings [24, 30]. LeadCare is also the main instrument described in 2013 guidelines by a CDC advisory committee for point of care BLL testing [31] and the only point of use device described in the 2011 WHO* brief guide to analytical methods for measuring lead in blood* [32]. LeadCare has been used in studies throughout the world, including the Zamfara, Nigeria lead poisoning epidemic [33, 34]. In the present study, all testing was conducted by a limited number of study-trained testers and adhered to manufacturer instructions for use, controls, and ambient conditions.

Skin and fingerstick-sample contamination is one possible explanation for the possible upward bias in LeadCare values (GFAAS samples were collected by venipuncture to provide the necessary amount of blood). The 2013 CDC guidelines did not specifically recommend use of lead-decontaminating skin wipes, but these are reported to be effective in addition to handwashing [35, 36]. We added them to our study protocol in a subsequent field project and the difference persisted between measurements by LeadCare in the field and GFAAS in the NIOEH laboratory (data not shown). Sample transport to and storage at the NIOEH laboratory are unlikely to affect sample quality, because blood lead is stable at ambient temperature and for up to a year with refrigeration or freezing [37, 38].

There is some reason for concern about possible* downward* bias in the study GFAAS analyses. GFAAS is a standard method for reference laboratory measurement of BLL. The study laboratory used conventional sample preparation and analytic procedures. However, quantitative accuracy was ascertained with in-house spiked controls and not with external certified reference (blood) material. The usual 90% recovery of spiked lead suggests a 10% downward bias with a nonblood control and uncertain accuracy with blood samples. Thus the discrepancy between GFAAS and LeadCare in this study does not necessarily discredit the field measurements.

Regardless of that, the venipuncture-lab retested samples generally affirmed the categorizations and clinical significance of the fingerstick-LeadCare results. We conclude that although there may be some imprecision and upward bias in the field-measured BLL values, these are not likely to substantially affect the relative distribution, categorically high magnitude, or clinical importance of lead intoxication in this village. Any clinical decisions about individual children would have been based on the confirmatory and more conservative GFAAS measurements. Nonetheless, there is a need for future scrutiny of this observed bias.

It was not possible to distinguish relative contributions to exposure by active recycling in the village, legacy contamination throughout the village, and recycling at centralized cooperative and private locations outside the village. The available evidence, however, suggested that all three provided ongoing sources of child lead exposure.

Active recycling within the village was almost certainly still a key source of lead exposure, at least for children (and adults) who lived in or near recycling households. Although only four screened children lived in such a household, all of them were in the highest BLL category, ≥45 *μ*g/dL. Surface lead levels were also highest at three XRF-measured households with active recycling operations. Furthermore, children's BLLs were higher with family involvement in recycling, most of which represented recycling away from the home. This suggested a substantial take-home pathway of lead from work at the centralized facilities or possibly the limited number of operations at nonsurveyed households. The importance of personal transport was supported by the contamination seen in household washing areas and on a limited sample of work clothes. Relatively high contamination on sleeping mats at school, compared to other school surfaces and soil, suggested transport from a nonschool source, possibly from home via children's clothing.

Lead exposure, however, did not appear limited to work sources and personal transport. Almost one-third of the tested children lived in households with no current or past family involvement in recycling, at the home or otherwise. Although they generally had lower BLLs than other children, 13 (of 31) had BLLs in the middle or highest categories used in this study. This suggested relatively widespread lead exposure, from either the small number of remaining recycling households or legacy contamination. In addition, although surface XRF measurements at a small sample of households revealed highest lead contamination at households with active recycling, measurements were substantially high at all sampled households, including those with no reported history of home recycling activities.

The BLLs measured in younger children did not clearly distinguish between one or another major source or pathway of lead exposure. However 40% of screened younger children having BLL ≥45 *μ*g/dL clearly signaled the urgent need for exposure reduction and remediation and if exposures could be controlled, medical management. Although there are no national child blood lead surveillance data for Vietnam, the observed BLLs are much higher than reported in population samples elsewhere in Southeast Asia [39–43]. Vietnam phased out lead in gasoline for road use in 2001 [44].

The XRF results indicated possible widespread lead contamination in village households, as well as lead transfer between work locations, homes, and the school. There are limitations to the usability of XRF values in this study. The small number of XRF-sampled households limited generalizability to other households. The use of an XRF “paint” mode to assess unpainted surfaces raised concerns that some fraction of detected surface lead might be embedded in the surface matrix and not readily mobilizable for human contact and uptake. However, the differences between measurement sites still had value for characterizing relative magnitudes of contamination. The present study provided useful information to guide possible mitigation efforts. However, the situation presented a daunting ethical and management challenge, given the magnitude and spatial extent of lead exposure, economic pressures to continue recycling, limited resources for remediation, no intervention funds in the study budget, child blood levels that warranted urgent attention, distance to providers with poisoning expertise, and the limited utility and relative contraindication of chelation treatment in the face of ongoing substantial exposure [21].

The 2012 stakeholder workshop identified major social, economic, and local and national political barriers to exposure reduction in Dong Mai village, established a need for additional exposure information (e.g., soil and surface lead measurements conducted after the workshop and reported here), identified proponents at provincial and national levels for possible interventions, and facilitated new connections with previously noninvolved groups, particularly the Vietnamese NGO, Centre for Environment and Community Development (CECoD), which was collaborating with the Blacksmith Institute on environmental health threats in craft villages. Ultimately, the connection between local and national government officials, CECoD, and the Blacksmith Institute led to a variety of intervention efforts at Dong Mai village. Those efforts and outcomes will be reported separately. The Vietnam NIOEH and UW research team have served as intervention advisors and evaluators.

## 5. Conclusions

This study adds to the body of evidence that battery recycling operations in developing countries can lead to clinically serious levels of lead intoxication in the adjoining community. Two other independent studies in Vietnam have demonstrated excessive child and adult lead exposure associated with battery recycling, in this same village and another craft village, both outside Hanoi [17, 18]. Given the proximity of two such villages near one urban center, it is very likely that there are comparable metalworking craft villages near other population centers in this large and rapidly developing country. The evolution in this study village from home-based operations towards centralized and larger-scale operations outside the village has not proved sufficient to eliminate the problems. There is a clear need to identify similar operations in Vietnam and to develop effective strategies that can manage, remediate, and ideally eliminate the pollutant health risks. These circumstances are undoubtedly not unique to Vietnam, and continued international efforts are needed to address the full scope of this serious health problem.

## Figures and Tables

**Table 1:** Child blood lead levels relative to child and household characteristics.

	Blood lead level^a^						Signif. (*p*)^b^
	10–29.9 *μ*g/dL		30–44.9 *μ*g/dL		≥45 *μ*g/dL		
	*n* = 36		*n* = 41		*n* = 32		
*Child*							
Age (years)							0.008
0–2	10	28%	9	22%	12	38%	
3–5	6	17%	13	32%	15	47%	
6–10	20	56%	19	46%	5	16%	
Sex (male)	13	36%	20	49%	18	56%	0.24
Symptoms							
Abdominal pain	13	36%	14	35%	6	19%	0.22
Constipation	19	53%	17	43%	16	50%	0.65
“Other” health problems	15	42%	25	63%	15	47%	0.17
Physical size							
Body mass index (BMI) (mean ± standard deviation)	15.4 ± 1.6		15.0 ± 1.8		15.5 ± 1.9		0.44
Height or length <5th percentile^c^							
Age 0–2 years	*n* = 10		*n* = 9		*n* = 12		
	5	(50%)	4	(44%)	3	(25%)	0.15
Age 3–5 years	*n* = 6		*n* = 13		*n* = 15		
	0	(0%)	4	(31%)	3	(20%)	0.30
Age 6–10 years	*n* = 20		*n* = 19		*n* = 5		
	7	(35%)	9	(47%)	1	(20%)	0.48
*Household lead recycling activities*	
Lead recycling at home							
Never	29	81%	31	76%	25	78%	0.02
Past; not currently	7	19%	10	24%	3	9%	
Currently	0	0%	0	0%	4	13%	
Family involvement in recycling							
Never	18	50%	5	12%	7	22%	0.002
Past; not currently	7	19%	10	24%	4	12%	
Currently	11	31%	26	63%	21	66%	
1 family member^d^	8	(73%)	17	(65%)	12	(57%)	0.67
>1 family member	3	(27%)	9	(35%)	9	(43%)	
*Home environment*							
Distance to current or past recycling site							
≤100 m to recycling facility	23	64%	25	61%	20	62%	0.97
≤100 m to burning of waste	12	33%	11	27%	4	13%	0.21
Battery casings at house (observed)							
Used outside	14	39%	17	41%	18	56%	0.30
Used inside	17	47%	17	41%	14	44%	0.88
Floor surface							0.85
Soil	1	3%	2	5%	1	3%	
Mats over bare soil	16	44%	19	46%	15	47%	
Finished (brick, cement, and tile)	15	42%	14	34%	12	38%	
Yard surface							0.07
Brick	17	47%	26	63%	22	69%	
Cement	15	42%	9	22%	6	19%	
Garden	17	47%	27	75%	20	56%	0.28

^a^Blood lead level determined by fingerstick blood sample and LeadCare II test instrument.

^b^Significance of between-column differences via chi-square, Fisher exact test, or one-way ANOVA.

^c^Height (or length) analyzed relative to age- and sex-adjusted lower 5th percentile, US CDC child growth standards.

^d^Percentages in parentheses show percent of subset.

**Table 2:** Multinomial logistic regression for child blood lead level categories, controlling for child and household effects.

Blood lead level (BLL)	Variable^a^	Odds ratio	95% confidence interval		Signif. (*p*)
30–44.9 *μ*g/dL	Age (years)				
	6–10	(1)^b^			
	3–5	1.9	0.5	7.5	0.35
	0–2	0.7	0.2	2.6	0.60
	Current family involvement in recycling	5.2	1.6	16.8	0.01
	Brick yard surface	3.5	0.9	13.7	0.07

≥45 *μ*g/dL	Age (years)				
	6–10	(1)			
	3–5	7.8	1.6	38.0	0.01
	0–2	4.1	1.2	14.3	0.02
	Current family involvement in recycling	7.6	1.9	30.0	<0.001
	Brick yard surface	4.6	1.0	20.6	0.05

^a^Current home recycling and proximity to recycling sites could not be included in the regression model due to zero values in reference cells.

^b^1 in parentheses denotes reference age category. The BLL category 10–29.9 *μ*g/dL is the reference category for each of the other BLL categories.

## Abbreviations

BLL: Blood lead level
CDC: US Centers for Disease Control and Prevention
CECoD: Vietnam Centre for Environment and Community Development
CLIA: US Clinical Laboratory Improvement Amendments
DALY: Disability-adjusted life year
EPA: US Environmental Protection Agency
GFAAS: Graphite Furnace Atomic Absorption Spectrometry
NIOEH: Vietnam National Institute of Occupational and Environmental Health
NIOSH: US National Institute for Occupational Safety and Health
NIST: US National Institute of Standards and Technology
OSHA: US Occupational Safety and Health Administration
ULAB: Used lead-acid battery
UW: University of Washington
WHO: World Health Organization
XRF: X-ray fluorescence.
